# Supplementary material for: MRI Assessed Placental Location as a Diagnostic Tool of Placental Invasiveness and Maternal Peripartum Morbidity
Source: Diagnostics (Basel). 2024 Apr 29;14(9):925. doi: 10.3390/diagnostics14090925 (PMC11083786; doi:10.3390/diagnostics14090925)
Supplement: Supplementary file 1 [file diagnostics-14-00925-s001.zip › sup file/Suppl Table S1.pdf]

Table 1. MRI protocol for placental evaluation for 1.5T magnet

Sequence	T2-SSTSE			T2-TSE					T1-TSEFS
Plane	Axial	Sagittal	Coronal	Axial	Sagittal	Coronal	Axial Oblique*	Coronal Oblique**	Axial
TR (ms)	510	532	568	6300	6300	6300	2500	2500	730
TE (ms)	80	80	80	90	90	90	90	90	6.9
NSA	1	1	1	2	2	2	2	2	1
Slice thickness/ Gap (mm)	5/1	5/1	5/1	3.5/1.2	3.5/1.2	4.5/1	4.5/1	4.5/1	5/1
Matrix	200x256	200x256	200x256	263x368	250x320	320x352	250x320	250x32	156x144
FOV A-P (mm)	320	375	345	320	360	329	280	280	380

* perpendicular to cervical axis (optional, when extrauterine spread is suspected)**parallel to cervical axis (optional, when extrauterine spread is suspected)

MRI indicates for magnetic resonance imaging; T2-SSTSE indicates for T2-weighted single-shot turbo spin echo; T2-TSE indicates forT2-weighted turbo spin echo; T1-TSEFS indicates forT1-weighted-turbo-spin-echo-fat-suppressed; TR indicates for time repetition; TE indicates for time echo; NSA indicates for number of signals acquired; FOV indicates for field of view; A-P indicates for anterior-posterior
