# Supplementary material for: MRI Assessed Placental Location as a Diagnostic Tool of Placental Invasiveness and Maternal Peripartum Morbidity
Source: Diagnostics (Basel). 2024 Apr 29;14(9):925. doi: 10.3390/diagnostics14090925 (PMC11083786; doi:10.3390/diagnostics14090925)
Supplement: Supplementary file 1 [file diagnostics-14-00925-s001.zip › sup file/Suppl Table S2.pdf]

Table 2. MRI protocol for placental evaluation for 3.0-T magnet

Sequence	T2W-MVXD			T2-TSE		T1W-TSE FS
Plane	Axial	Sagittal	Coronal	Axial Oblique*	Coronal Oblique**	Axial
TR (ms)	4345	4755	4666	4807	4807	723
TE (ms)	100	100	100	90	90	8.0
NSA	1	1	1	1	1	1
Slice thickness/Gap (mm)	5/0.5	5/1.2	5/0.5	3.0/0.0	3.0/0.0	5.0/1.2
Matrix	380x380	380x380	400x400	290x310	290x310	252x277
FOV A-P (mm)	320	380	380	230	230	350

*perpendicular to cervical axis; **parallel to cervical axis

MRI indicates for magnetic resonance imaging; T2W-MVXD indicates for T2-weighted-MultiVaneXD; T2-TSE indicates for T2-weighted turbo spin echo; T1-TSEFS indicates for T1-weighted-turbo-spin-echo-fat-suppressed; TR indicates for time repetition; TE indicates for time echo; NSA indicates for number of signals acquired; FOV indicates for field of view; A-P indicates for anterior-posterior
